# Prevalence and incidence of domestic violence during pregnancy and associated risk factors: a longitudinal cohort study in the south of Sweden

**DOI:** 10.1186/s12884-016-1017-6

**Published:** 2016-08-17

**Authors:** Hafrún Finnbogadóttir, Anna-Karin Dykes, Christine Wann-Hansson

**Affiliations:** 1 Faculty of Health and Society, Department of Care Science, Malmö University, Malmoe, Sweden; 2 Department of Health Sciences, Medical Faculty, Lund University, Lund, Sweden

**Keywords:** Domestic violence, Pregnancy, Prevalence, Incidence, Risk factors

## Abstract

**Background:**

Domestic violence during pregnancy is not only a severe public health issue that jeopardizes maternal and foetal health but also violates human rights. The *aim* was to explore the prevalence and incidence of domestic violence among pregnant women, in the southwestern region of Scania in Sweden, and their experience of a history of violence. In addition, to explore the association between domestic violence during pregnancy and possible risk factors.

**Methods:**

This is a longitudinal cohort-study including pregnant women ≥18 years of age, registered at antenatal care when pregnant. A cohort of 1939 pregnant women answered Questionnaire I (QI) in gestational week 13 (mean 12.8 week, SD 5.11). Response rate of Questionnaire II (QII) in gestation week 34 (mean 33.9 week, SD 2.2) was 78.8 % (*n* = 1527). Statistical analysis was descriptive statistics, logistic regression and multiple regression with Odds ratios (OR) and 95 % confidence intervals (95 % CI).

**Results:**

Both QI and QII were completed by 77.8 % (*n* = 1509) of the women and 44.3 % (*n* = 668) reported a lifetime experience of abuse irrespective of type, severity or perpetrator. Also, 5.1 % (*n* = 77) reported some experience of abuse past year. Prevalence of domestic violence during pregnancy was 2.0 % (*n* = 29) and the incidence was 7.3 new cases per 1000 women. The strongest risk factor for domestic violence, during early and late pregnancy, was *history of violence* whereby all women who disclosed exposure had also reported *history of violence* (*p* < 0.001). To be single/living apart gave 8.4 times more risk associated with domestic violence during pregnancy (AOR 8.4; 95 % CI: 2.2–32.6). Having several symptoms of depression and lack of sleep gave 3.8 times more risk respectively (AOR 3.8; 95 % CI: 1.1–13.6) and (AOR 3.8; 95 % CI 1.1–12.9).

**Conclusions:**

Pregnant women with a history of violence as well as being single/living apart and/or having several symptoms of depression during pregnancy should be alerts for clinical working midwives and obstetricians. Further, this is important knowledge for health care providers to develop or upgrade guidelines and plans of action for pregnant women exposed to violence.

## Background

Domestic violence (DV) during pregnancy is not simply a severe public health issue that jeopardizes maternal and foetal health, [1–8] but also a violation human rights [6] and according to the Swedish penal code a criminal act [9]. The World Health Organization (WHO) has reported that more than 90 % of the abused pregnant women are abused by the biological father of the child the woman was carrying [10]. There is a large variation in the prevalence of reported violence, which can be explained by differences in the material, definitions and methodologies used, as well as the context [11–14]. However, longitudinal studies that shows the incidence of DV during pregnancy are rare. Further there is a lack of evidence about potential risk factors that are associated with the incidence of DV during pregnancy.

A meta-analysis of 92 independent studies completed in 23 countries (Sweden included) published in 2013, revealed an average prevalence of DV during pregnancy of 19.8 % where 28.4 % of these were characterised as emotionally based, 13.8 % as physical violence and 8.0 % were sexual abuse [14]. The overall prevalence of DV during pregnancy in developed countries was reported as 13.3 % in comparison to 27.7 % in the less developed countries [14]. In Sweden the prevalence of abuse during pregnancy has previously been reported as 1.0 % DV during early pregnancy (mean 12.8 week, SD 5.11) [11], 1.3 % perpetrated by a close acquaintance or relative during or shortly after pregnancy [12], and 4.3–14.5 %, depending on the severity of the violence, was perpetrated by a current or ex-partner [13].

In a meta-analysis of 55 independent studies, the strongest predictor for DV among pregnant women disclosed as having a history of violence [14]. In addition, it has been reported in a study from six countries in northern Europe that a history of abuse is common among pregnant women [15] which was also revealed in our earlier study [11]. Other identified risk factors for DV among pregnant women are being single, having a lower standard of education as well as low socioeconomic status, an unintended pregnancy and when the perpetrator of the abuse misused alcohol [14]. Another systematic review and meta-analysis showed that high levels of anxiety, symptoms of perinatal depression and posttraumatic stress disorder (PTSD) were significantly associated with the experience of DV during a woman’s lifetime, including while pregnant [7].

The health and well-being of a mother-to-be is also reflected in the offspring’s health and the pregnancy outcome. A systematic review of 30 studies, disclosed that pregnant women exposed to DV are almost 1.5 times more likely to have a preterm baby as well as delivering a low-birth-weight baby [8]. Abuse of pregnant women indirectly affects (i.e. increased risk of various psychological and physical health problems) or directly (i.e. abrupt trauma to the stomach) the morbidity and mortality of both the mother and the offspring [1–5].

An absolute condition for a pregnant woman surviving the abuse is that it is identified early in order to get adequate help in the form of support and care. A Cochrane review supports the fact that disclosure of violence increases significantly, when the question of exposure to violence is addressed, especially at Antenatal Care (ANC) [16]. In late 2014, The National Board of Health and Welfare in Sweden recommended that all women received by a midwife at an ANC should be asked about any experience of violence they had [17]. However, it can vary locally from county to county as to how and to what extent this sensitive matter is addressed. It is the Healthcare providers’ responsibility to develop procedures for how and when the issue of violence is to be discussed with the mother-to-be. As well as to develop procedures for the staff to follow when they detect a woman who has been exposed to violence. Further, it is also the health care provider’s responsibility to ensure that the staff are working in accordance with these procedures. Today, there are not only public, but also private clinics for pregnant women in Sweden. In the year 2014, almost 80 % of all pregnant women in Sweden were asked by their midwife, at their ANC, if they had any experience of violence [18]. Obstacles to early recognition of the problems of violence can be the lack of local guidelines as well as lack of professional support and overall, the problem of the midwives themselves if they lack knowledge or confidence [19]. Added to this is also the fear of the possible reaction of the perpetrator (ibid). Also, it is fundamental to have prepared strategies to follow up and refer a survivor of violence to help in order to get positive effects for the pregnant woman and her child’s health and to decrease or eliminate the violence [16].

The literature appears to be inconsistent across cultures concerning whether pregnancy is a time of protection or risk to be exposed to DV [20]. In our previous report which is the first part of this whole project, the recruitment of pregnant women was undertaken at 17 ANC’s, both public and privately driven [11]. All violence-exposed women also reported a history of violence regardless of type or level of abuse. There was a seven-fold risk for having several symptoms of depression if exposed to violence (ibid). As there is a shortage of reports about the incidence of DV during pregnancy and related risk factors, there was a need to explore this subject area to get a better picture of the magnitude of this problem.

The *aim* was to explore the prevalence and incidence of domestic violence among pregnant women in the southwestern region of Scania in Sweden, as well as their experience of a history of violence. In addition, to explore the association between domestic violence during pregnancy and possible risk factors.

## Methods

### Design and setting

This cohort study has a longitudinal design and is the second report from the project entitled *“Pregnant women and new mother’s health and life experience”* where the data collection was performed in the southwest area of Scania in Sweden. Setting, participants and recruitment to the study are explained in detail elsewhere [11]. The catchment area is characterised by multicultural diversity.

### The characteristics of participants

The inclusion criteria were women ≥18 years of age, registered at an ANC when pregnant and who could understand and write Swedish or English. Almost 80 % of the participants had Sweden as their country of origin and the remaining pregnant women were born in 93 different foreign countries [11].

### Process of recruitment

**Fig. 1:**
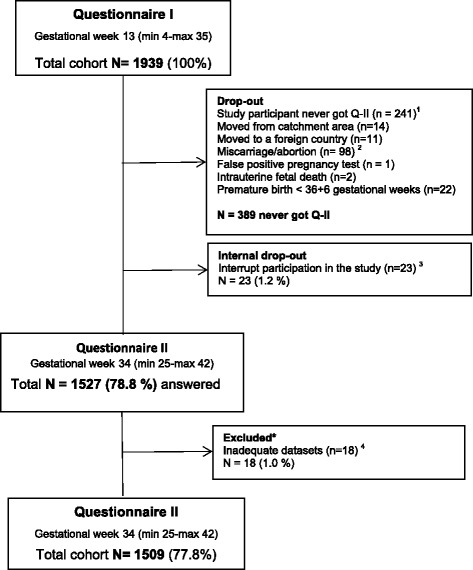
Flowchart over received answers in Questionnaire I and II. * No one reported abuse during pregnancy. 1) The midwives forgot to give the participants questionnaire II (*n* = 239), Missing consent (*n* = 2). 2) Spontaneous and legal abortions (*n* = 84), missed abortions (*n* = 4), spontaneous and legal abortions due to malformations or for social reasons >18 gestational weeks (*n* = 10). 3) No explanation or did not understand the violence questions well enough or had difficulties with the language (*n* =20). Also, participant too stressed to stay to complete the questionnaire (*n* = 3). 4) Failure with the verification or wrongly coded

The pregnant women received individual verbal and written information about the study from their midwife and were invited to answer the questionnaires in a private place at their ANC facility. If any of the participants asked for help, it was offered to them by health professionals. Power calculation performed by a statistician showed that at least 2000 participants were required for statistical calculations to answer by 98 % certainty at least 2.5 % prevalence of DV. The participants were recruited in *early pregnancy* between March 2012 and September 2013 and requested to answer Questionnaire I (QI). Further, the data collection was continued with Questionnaire II (QII) and was completed at the beginning of April 2014. Of the total cohort of 1939 pregnant women who took part in the study and answered QI in *early pregnancy*, there were 78.8 % (*n* = 1527) who answered QII in *late pregnancy*. In total, 389 women never received QII. However, the complete dataset from QI and QII totalled 77.8 % (*n* = 1509) of the 1939 participants (Fig. 1).

### Questionnaires

All data was based on self-administrated questionnaires (QI and QII) completed in as private a place as possible at the ANC’s. By the time they answered QII, the participants were familiar with the questions about any experience of violence. The questions were reduced from 122 in QI to 93 in QII. Some background questions and the Sense of Coherence scale (SOC-13) [21] was excluded in QII. The main instrument used, NorVold Abuse Questionnaire (NorAQ) has shown good reliability, validity and specificity regarding the abuse variables [22] and is well described in previous study [11]. Questions about psychological, physical and sexual abuse are included in the study as well as the severity of the violence. In addition, one additional question, modified from the Abuse Assessment Screen (AAS) used to investigate current abuse during pregnancy, was added to the NorAQ (ibid). The instruments; Edinburgh Postnatal Depression Scale (EPDS) [23], used during pregnancy (EDS) [24] as well as the Alcohol Use Disorders Identification Test (AUDIT) [25] are included in the questionnaires and described in detail elsewhere [11].

### Definitions

The definition of domestic violence used in this study is in agreement with the WHO’s definition [26], as physical, sexual or psychological, or emotional violence, or threats of physical or sexual violence that are inflicted on a pregnant woman by a family member, i.e. an intimate male partner, marital/cohabiting partner, parents, siblings, or a person very well known to the family, or a significant other, (i.e. former partner) when such violence often takes place in the home. Further, according to Swahnberg et al. [22] definitions for severity of abuse, which classifies abuse as mild, moderate or severe and also the type of abuse used are well described in earlier study [11]. A *history of violence* is defined as a lifetime experience of emotional, physical or sexual abuse occurring during childhood (<18 years), adulthood (≥18 years) or both, regardless of the level of abuse or the perpetrator’s identity, in accordance with the operationalization of the questions in the NorAQ.

### Classification of the variables

In this study we have used the Same classification of variables as used in earlier study [11] which were as follows; *Age* was classified and dichotomized as 18–34 and ≥35 years, *Language* as a foreign language spoken at home or Swedish (solely). *Educational status* as a *low educational status*, i.e. basic schooling versus a *high educational status* such as high school or university. *Cohabiting status* was classified as being single/living apart, or as a common law spouse/married. *Employment status* was dichotomized as employed (including parental leave and studying) or unemployed (including long-term illness). *Financial distress* was dichotomized as “no” (no problem) or “yes” (serious financial distress). Maternal characteristics concerning *body mass index* (BMI) were calculated from maternal weight and height before the pregnancy and classified according to WHO’s definition [27] as underweight (<18.5), normal weight (18.50–24.99), overweight (≥25–29.99), and obese (≥30) and dichotomized as under-/normal weight or overweight/obese. *Smoking* was dichotomized as “yes” (if the woman was a daily smoker or smoked at some point during pregnancy) and “no” (never smoked or stopped before pregnancy). The use of *wet tobacco* was dichotomized as “yes” (if the woman was a daily user at some point during pregnancy) and “no” (never used *wet tobacco* or ceased before pregnancy), further *smoking and/or using wet tobacco* was dichotomized as “yes” versus “no”. *Alcohol use* was dichotomized as “yes” or “no”. *Unintended pregnancy* was dichotomized as “yes” or “no”. *Abortion/miscarriage* was classified as “no”, “miscarriage”, “abortion” or both “miscarriage/abortion” and dichotomized as to no or miscarriage/abortion. *Self-reported health* was dichotomized as poor health versus rather good health. Sleep, was dichotomized as *Lack of sleep* versus *adequate sleep.*

### Statistical methods

Descriptive statistics were used to show the prevalence and severity of a lifetime experience of any type and level of abuse. OR and 95 % CI were calculated for the crude associations between possible risk factors and ‘DV during pregnancy’, with ‘DV during pregnancy’ as a dependent variable for bivariate logistic regression. For the purpose of bivariate logistic regression, a variable for depression was computed based on EDS scores, i.e. symptoms of depression during pregnancy, whereby an optimal cut-off of ≥13 was chosen as representing the presence of symptoms of depression [24]. The EDS score was computed only for those responding to all ten questions (missing = 102). In order to analyze the association between SOC score and exposure to ‘DV during pregnancy’, the SOC-scale was dichotomized utilizing the first quartile of the distribution as a cut-off value (SOC ≤64 and SOC >64) [28]. The SOC score was only computed for those responding to all thirteen items (missing = 96). Multiple logistic regression was performed in order to evaluate the influence of variables that were significant in the bivariate logistic regression with ‘DV during pregnancy’ as a dependent variable; the multiple logistic regression analyses were thus step-wise adjusted (forward selection) for; Single/living apart, EDS ≥13, Low SOC-score, Lack of sleep, Financial distress, Low educational status, Unintended pregnancy, and Age. Statistical significance was accepted at *p* < 0.05. Statistical analyses were performed using the Statistical Package for Social Sciences (SPSS) version 22.0 for Windows.

## Results

The response rate for QII was 78.8 % (*n* = 1527) of the total cohort (*n* = 1939) of women who were recruited in *early pregnancy* during gestational week 13 (mean 12.84, SD 4.11, min 4-max 35) and had completed QI. QII was answered during *late pregnancy* or during gestational week 34 (mean 33.9 week, SD 2.2, min 25-max 42). Both QI and QII were completed by 77.8 % (*n* = 1509) of the participants and 44.3 % (*n* = 668) of them reported a lifetime experience of abuse irrespective of type, severity or perpetrator. Also, 5.1 % (*n* = 77) reported some experience of abuse during the past year i.e. during pregnancy as well as some months before the pregnancy being known (Table 1).

**Table 1:** Type and severity of lifetime abuse: in Questionnaire I, Questionnaire II, Questionnaire I and II

Type and severity of abuse	Questionnaire I	Questionnaire II	Questionnaire I + II^b^
	Early pregnancy	Late pregnancy	
	n (%)	n (%)	n (%)
	1928 (100)	1497 (100)	1509 (100)
*Missing*	11^a^	30^a^	
Lifetime experience of abuse^c^	761 (39.5)	562 (36.8)	668 (44.3)
Any abuse during the past year	84 (4.3)	38 (2.5)	77 (5.1)
Lifetime of emotional abuse	374 (19.5)	257 (16.8)	335 (22.2)
*Mild*	307 (16.1)	221 (14.5)	291 (19.3)
*Moderate*	187 (9.8)	123 (8.1)	175/11.6)
*Severe*	203 (10.6)	135 (8.8)	192 (12.7)
Any emotional abuse during the past year	61 (3.1)	28 (1.8)	56 (3.7)
Lifetime of physical abuse	561 (29.3)	417 (27.3)	514 (34.1)
*Mild*	529 (28.0)	399 (26.1)	493 (33.5)
*Moderate*	203 (10.7)	171 (11.2)	214 (14.5)
*Severe*	127 (6.7)	89 (5.8)	118 (8.0)
Any physical abuse during the past year	36 (1.9)	13 (0.9)	31 (13.0)
Lifetime of sexual abuse	302 (15.7)	218 (14.3)	265 (17.8)
*Mild* ^*d*^	49 (2.6)	37 (2.4)	52 (3.6)
*Mild* ^*e*^	208 (11.0)	169 (11.1)	217 (14.9)
*Moderate*	212 (10.9)	166 (10.9)	199 (13.5)
*Severe*	144 (7.4)	94 (6.2)	118 (8.0)
Any sexual abuse during past year	2 (0.1)	5 (0.3)	5 (0.3)

^a^Not answered the questions about violence

^b^Self-reported in Questionnaire I or II or both

^c^Any type of self-reported abuse during lifetime irrespective perpetrator

^d^Emotional or sexual humiliation

^e^No genital contact

### Prevalence and incidence of DV during pregnancy

Table 2 shows the prevalence and incidence of DV during pregnancy. The prevalence of DV during pregnancy (solely) irrespective of type or severity was reported to be 2.0 % (*n* = 29). The incidence of DV during pregnancy was 7.3 new cases per 1000 pregnant women and there were 11 new women who reported DV during pregnancy. In QI 1.0 % (*n* = 18) of the participant women reported DV in *early pregnancy* and in QII there were 1.1 % (*n* = 17) of the women who reported that during *late pregnancy* they were exposed to DV (Table 2). Of the 18 women reporting DV during pregnancy in QI, 12 of them did not report DV during pregnancy in QII. This can be explained as follows; three had miscarriages/abortion, one moved from the catchment area, four did not fill-in the QII and four did not report any DV during pregnancy in QII (this information is exclusively presented in the text).

**Table 2:** Self-reported prevalence and incidence of DV during pregnancy^**a**^

Characteristics	Questionnaire I	Questionnaire II	Prevalence of DV during pregnancy ^b^	Incidence ^d^ of DV during pregnancy
	Early pregnancy	Late pregnancy		
	n (%)	n (%)	n (%)	n (%)
Total cohort	1939 (100)	1527 (100)	1939 (100)	1509 (100)
In the analysis	1928 (99.4)	1497 (98.0)	1467 (75.7)	1497 (99.2)
*Missing* ^c^	11 (0.6)	30 (2.0)	472 (24.3)	12 (0.8)
Emotional abuse	15 (0.8)	13 (0.9)	24 (1.6)	9 (6.0)
Physical abuse	7 (0.4)	8 (0.5)	11 (0.7)	3 (2.0)
Sexual abuse	2 (0.1)	2 (0.1)	2 (0.1)	0 (0.0)
Total of any type of abuse	18 (1.0)	17 (1.1)	29 (2.0)	11 (7.3)

^a^ Some women may report more than one type of violence

^b^ Self-reported at least once in QI or QII or both questionnaires

^c^ Excluded in the analysis, because the questions about violence were not answered

^d^ Numbers and incidence pr 1000 women

### Association between possible risk factors and exposure to DV during pregnancy

The strongest risk factor for DV being reported during late pregnancy was *a history of violence* whereby all of the women (*n* = 17) who had disclosed exposure to DV during pregnancy had also reported ‘a history of violence’ (*p* < 0.001). Women with *a low educational status* were 3.1 times more likely to report being exposed to DV during pregnancy (*p* = 0.016). Women living *Single/living apart* were 17.9 times more likely to report exposure to DV during pregnancy (*p* < 0.001). Further, women in a situation of *financial distress* were 3.7 times more likely to be exposed to DV during pregnancy (*p* = 0.014). Also, women with *an unintended pregnancy* were 2.8 times more likely to be exposed to DV during pregnancy (*p* = 0.040). Those who reported *lack of sleep* during the last year, to such an extent that they had problems coping with their daily life, were 9.6 times more likely to be exposed to DV during pregnancy (*p* < 0.001). Women having an EDS score ≥13 indicating the presence of several symptoms of depression were 15.8 times more likely to be exposed to DV during pregnancy (*p* < 0.001). Lastly, women having a low score on the SOC-scale, indicating an inability to use their own resources to maintain and improve their health in stressful situations were 9.9 more likely to be exposed to DV during pregnancy (*p* < 0.001) (Table 3).

**Table 3:** Association between possible risk factors and DV during late pregnancy (*N* = 1509)

Independent variable	n (%)	DV reported during late pregnancy n (%)	OR 95 % CI	*P*-value (two-tailed)
History of violence^a^	668 (44.3)	17 (2.6)	-	<0.001
Age ≥35	269 (18.1)	2 (0.8)	0.6 (0.1–2.7)	NS
Multiparae	760 (54.3)	11 (1.5)	1.9 (0.6–5.4)	NS
Low educational status	481 (31.9)	10 (2.1)	3.1 (1.2–8.2)	0.016
Unemployed	74 (4.9)	2 (2.9)	2.7 (0.6–12.2)	NS
Foreign language	357 (23.7)	6 (1.7)	1.8 (0.7–4.9)	NS
Single/living apart	50 (3.3)	6 (12.2)	17.9 (6.4–50)	<0.001
Financial distress	705 (46.7)	13 (1.9)	3.7 (1.2–11.5)	0.014
Alcohol consumption	330 (22.1)	4 (1.2)	1.1 (0.4–3.4)	NS
Smoking/using wet tobacco	300 (20.1)	5 (1.7)	1.7 (0.6–4.8)	NS
Overweight/obese	378 (26.0)	7 (1.9)	2.0 (0.8–5.3)	NS
Unintended pregnancy	246 (16.5)	6 (2.4)	2.8 (1.01–7.5)	0.040
Miscarriage/abortion	91 (6.2)	2 (2.2)	2.1 (0.5–9.1)	NS
Self-reported poor health	67 (4.6)	1 (1.5)	1.4 (0.2–10.7)	NS
Lack of sleep	107 (7.2)	7 (6.7)	9.6 (3.6–25.8)	<0.001
EDS ≥13	115 (8.2)	9 (8.0)	15.8 (5.8–43.4)	<0.001
SOC Low score	364 (25.4)	13 (3.7)	9.9 (3.2–30.7)	<0.001

Statistical significant is accepted by *p* < 0.05

^a^ All (*n* = 17) reported history of violence and therefore OR with 95 % CI not shown

Table 4. Here, the resulting outcomes in Table 3 were checked for the following variables; single/living apart, EDS ≥13, low SOC score, lack of sleep, financial distress, low educational status, unintended pregnancy and age. Single/living apart remained significant (*p* = 0.002) and had 8.4 times risk associated with DV during pregnancy, EDS scores ≥13 and lack of sleep had 3.8 times the risk respectively (*p* = 0.04, *p* = 0.03).

**Table 4:** Association between possible risk factors and exposure to DV during pregnancy in late pregnancy (*n* = 17)

Variables	Model I	Model II	Model III	Model IV	Model V	Model VI	Model VII	Model VIII
	OR (95 % CI)	OR (95 % CI)	OR (95 % CI)	OR (95 % CI)	OR (95 % CI)	OR (95 % CI)	OR (95 % CI)	OR (95 % CI)
Single/living apart ^a^	18.0 (6.4–51.0)	9.2 (2.9–29.6)	7.9 (2.4–25.5)	8.4 (2.5–28.0)	8.5 (2.4–29.8)	7.2 (2.0–26.2)	8.2 (2.1–32.0)	8.4 (2.2–32.6)
EDS ≥13 ^b^		9.9 (3.3–29.4)	5.4 (1.7–17.5)	3.5 (1.0–12.6)	3.5 (1.0–12.7)	3.6 (1.0–12.7)	3.7 (1.0–13.2)	3.8 (1.1–13.6)
Low score SOC ^c^			3.8 (1.0–13.6)	3.4 (0.9–12.3)	3.4 (0.9–12.7)	3.2 (0.9–12.0)	3.3 (0.9–12.2)	3.3 (0.9–12.3)
Lack of sleep ^d^				3.8 (1.1–12.5)	3.8 (1.1–12.6)	4.0 (1.2–13.2)	3.9 (1.2–13.3)	3.8 (1.1–12.9)
Financial distress ^e^					1.0 (0.3–3.7)	0.8 (0.2–3.2)	0.8 (0.2–3.2)	0.8 (0.2–3.2)
Low educational status ^f^						2.0 (0.6–6.4)	2.2 (0.7–7.2)	2.2 (0.6–7.1)
Unintended pregnancy ^g^							0.7 (0.2–2.7)	0.8 (0.2–2.8)
Age ^h^								1.2 (0.2–6.0)

^a^ Single/living apart versus cohabiting (reference category)

^b^ EDS ≥13, indicating having a risk of depression versus not ≤13 (reference category)

^c^ Low score SOC indicating inability to use their own resources to maintain and improve their health in stressful situations versus medium-high score (reference category)

^d^ Lack of sleep versus adequate sleep (reference category)

^e^ Financial distress versus no financial distress (reference category)

^f^ High school or less versus more than high school (reference category)

^g^ Unintended versus intended pregnancy (reference category)

^h^ Age 18–34 versus age ≥35 years (reference category)

## Discussion

The present study showed the prevalence of DV as being 2 % in women during their current pregnancy (solely). This means that at least 180 pregnant women in the catchment area are exposed to DV during pregnancy annually (calculated on 9000 births in the catchment area or at one university hospital with two delivery apartments). This results in a twofold prevalence when compared with our earlier study [11] were the women answered QI in early pregnancy. This can indicate that DV during pregnancy is not only a continuum of pre-existing DV [10, 11], but likely to increase over time [29], which is also in accordance with the normalizing process (where the violence has become a natural part of the relationship) [30] and noted in an earlier study by Finnbogadóttir et al. [31]. However the true prevalence can be difficult to determine of many reasons, as for example fear concerning abuse escalation if the perpetrator would be made aware through disclosure [32], as well as any sense of shame over the situation the violence-exposed woman finds herself in [19, 31, 33–35]. In addition, in our prior study [11] it was indicated that the prevalence of DV during early pregnancy might be underestimated due to reported higher prevalence of lifetime physical abuse performed by an actual partner as well as due to selection or non-respondent bias. In the current study there is also an indication that the prevalence of DV might be underestimated due to that 5.1 % of the pregnant women reported experience of some abuse over the past year. This involves experience of abuse perpetrated some months prenatal as well as during pregnancy. This is also in agreement with earlier research from countries in northern Europe where the same questions from the same instrument NorAQ had been used; Norway 5 % [36] and 3.7 % respectively [15], Iceland 3.3 %, Belgium 3.0 %, Denmark 3.3 %, Estonia 6.5 % and Sweden 3.0 % (ibid). In the present study the prevalence of DV during pregnancy appears to be realistically perceived in a global perspective, because the prevalence in developed countries compared to less developed countries (more violence-tolerant societies) has shown to be lower [14, 37]. Still, the true prevalence is difficult to estimate but can approach the prevalence level indicated in reality studies performed in different contexts from different countries.

In addition, the present study showed the incidence of DV during pregnancy to be 7.3 new cases per 1000 pregnant women or at least 65 new violence-exposed pregnant women annually (calculated on 9000 births). It has been suggested that the pregnancy per se can trigger violence [38, 39] as well as that the violence during pregnancy may be only a continuum of previous violence [10, 11]. This was also supported by the current study, where all of the women who were exposed to DV during pregnancy also reported a history of violence. However, the literature is inconsistent regarding this point, but it has been pointed out that cultural factors may be important determinators [37]. Corresponding, studies have shown that repeated questioning about the experience of violence increases the possibility that the violence-exposed woman admits her vulnerability [12, 40]. The complexity of the topic and the pre-existing strain in the relationship, as well as new demands made during pregnancy, may also explain new cases of abuse during pregnancy. Further, in many cases the survivor wants the relationship to work [31, 41] and therby does not disclose the ongoing abuse. Correspondingly, violence-exposed pregnant women are prone to stay in a dysfunctional relationship in order to protect their unborn baby and are not willing to leave the relationship for reasons of abuse, and also if their self-esteem and self-respect has been lost and they experience that they feel drained of their energy [31]. Altogether this is extremly important information for every caregiver when meeting the violence exposed pregnant women and can guide them in how their actions/practises to help and support should be planned for each individual woman.

History of violence was separately, the strongest risk factor for being exposed for DV during pregnancy. This is congruent with previous research [11, 14]. Further, our data suggests that a history of violence is a common occurrence among pregnant women since more than four out of ten (44.3 %) women in the study had experienced a history of violence. This is also verified by earlier research [11, 15]. Indeed women with a history of violence in their relationship may be at increased risk for DV during pregnancy [11, 14] therefore it’s of utmost importance to approach the matter with the pregnant women at the ANC’s as well as to have the possibility to give the survivor first line support. This is in accordance with the WHO’s clinical guidelines from 2013 as well as the national recommendations in Sweden [17]. Furthermore, when a midwife or obstetrician discovers a violence-exposed pregnant woman in Sweden they are obliged to report their findings to the social services if there are other children in the family and/or in order to protect the unborn baby [42–44]. There can be a reluctance among women to disclose their current situation regarding DV due to their fear that their other children might be taken away from them. In our former study, this misapprehension was clearly pointed out by one of the interviewed midwives who had experience of an immigrant woman who thought the authorities would take her other children away from her if the violence was revealed [19]. However, in the same study the midwives also expressed their fear of retaliation against themselves or their families if they should report suspected DV to the authorities if the perpetrator was known to have shown very aggressive behaviour (ibid). Therefore, it’s extremely important to have a clear and safe plan of action for the violence-exposed pregnant women as well as for all of the health care personnel at the ANC’s.

The present study also revealed the following strong predictors for pregnant women to be exposed to DV during pregnancy on the grounds of their being; ‘single/living apart’ which is supported by earlier research [14] as well as the ‘presence of several depressive symptoms’ which is correspondingly supported by systematic review and meta-analysis [7]. Having a ‘lack of sleep’, which is also supported by earlier studies [11, 31]. Lack of sleep is one of the signs for PTSD as well as depression [45]. To be single/living apart as well as suffering from a lack of sleep during the last year to such an extent that the pregnant woman has problems coping with her daily life should be an alarm signal to the midwife and other health care providers indicating strong predictors for DV during pregnancy. Moreover, the presence of several depressive symptoms detected in early pregnancy [11], as well as in late pregnancy, [7] can also indicate that the woman is exposed to DV during pregnancy. Hypothetically, a pregnant violence-exposed woman who reports herself as being single/living apart and exposed to DV may have recently left the abusive relationship during pregnancy. Another possible scenario is that the perpetrator she has already left stalks her and will not leave her alone.

### Strength and weakness in the study

The current cohort study with its longitudinal design based on prospectively collected data allowed the comparison of violence exposed and non-violence exposed pregnant women during the same time-period, which is considered as a strength for the study as well as offering the possibility to explore both prevalence and incidence of violence. Further, using validated instruments in the questionnaires [21–23, 25, 46, 47] where the main instrument was previously used in a multicounty study, [48] and also validated within a Swedish population, [22] is also considered as a strength. Initially, power calculation was performed and the number of participants was slightly underpowered with 1939 instead of 2000. The results might potentially be biased due to selection. This is because the recruitment took more time than expected due to a high work load among the recruiting midwives and therefore the selection became nonconsecutive, but random. However, we do not find any reason to believe that systematic selection bias occurred. The strength of the current study is the sample size in QI (*n* = 1939) and the satisfactory number of answers of 78.8 % in QII. The fact is, only 1.5 % of those (*n* = 1550) who received the QII constitutes an internal drop-out (Fig. 1). However, due to an administration failure the recruiters (the midwives) failed to give the second questionnaire to 239 participants (12.3 %) which may reflect how strained their working situation was during the participant recruiting period. Therefore, unfortunately the prevalence and incidence of DV during pregnancy may be underestimated. The results from the study are also limited to those who fulfilled the inclusion criteria ≥18 years, registered at an ANC when pregnant, and who understood and could write Swedish or English.

## Conclusions

With the result that at least 2 % of the pregnant women in this study were enduring DV, there is a clear need to address the situation of this vulnerable group of women in order to take steps to improve maternal and child health. Midwives and obstetricians who meet women with a history of violence at ANC should be aware of the possibility of additional risk factors in the anamneses. Such as, being single/living apart, having a long term problem with sleeping and/or having several symptoms of depression during pregnancy. All these factors should be indicators to alert health care providers who can use this knowledge to develop or upgrade guidelines and plans of action for helping pregnant women who are exposed to violence.

## Abbreviations

AAS, abuse assessment screen; ANC, antenatal care; AUDIT, alcohol use disorders identification test; BMI, body mass index; CI, confidence intervals; DV, domestic violence; EDS, Edinburgh depression scale; EPDS, Edinburgh postnatal depression scale; NorAQ, NorVold abuse questionnaire; QI, questionnaire I; QII, questionnaire II; OR, odds ratios; SOC-13, sense of coherence scale-short form; WHO, World Health Organization

## References

[1] Boy A, Salihu HM (2004). Intimate partner violence and birth outcomes: a systematic review. Int J Fertil Womens Med.

[2] Jasinski JL (2004). Pregnancy and domestic violence: a review of the literature. Trauma Violence Abuse.

[3] Sharps PW, Laughon K, Giangrande SK (2007). Intimate partner violence and the childbearing year: maternal and infant health consequences. Trauma Violence Abuse.

[4] Shoffner DH (2008). We don’t like to think about it: intimate partner violence during pregnancy and postpartum. J Perinat Neonatal Nurs.

[5] Chambliss LR (2008). Intimate partner violence and its implication for pregnancy. Clin Obstet Gynecol.

[6] World Health Organization; Violence against women. Intimate partner and sexual violence against women. Facta sheet No 239 [http://www.who.int/mediacentre/factsheets/fs239/en/]. Accessed 8 July 2015.

[7] Howard LM, Oram S, Galley H, Trevillion K, Feder G (2013). Domestic violence and perinatal mental disorders: a systematic review and meta-analysis. PLoS Med.

[8] Shah PS, Shah J, Knowledge Synthesis Group on Determinants of Preterm/LBW Births (2010). Maternal exposure to domestic violence and pregnancy and birth outcomes: a systematic review and meta-analyses. J Womens Health.

[9] Regeringskansliets rättsdatabaser, Brottsbalk 1962:700. http://www.notisum.se/rnp/SLS/LAG/19620700.htm#K4P4S1 4 kap. Lag (2013:367) 4 a § (in Swedish). InEnglish: Government Office, Swedish Statute Book.Penal code 1962:700 Law (2013:367). 2013, 4 a §. Accessed 9 Aug 2016.

[10] World Health Organization (WHO) (2005). WHO Multi-country Study on Women’s Health and Domestic Violence against Women. Initial results on prevalence, health outcomes and women’s responses.

[11] Finnbogadottir H, Dykes AK, Wann-Hansson C (2014). Prevalence of domestic violence during pregnancy and related risk factors: a cross-sectional study in southern Sweden. BMC Womens Health.

[12] Stenson K, Heimer G, Lundh C, Nordstrom ML, Saarinen H, Wenker A (2001). The prevalence of violence investigated in a pregnant population in Sweden. J Psychosom Obstet Gynaecol.

[13] Hedin LW, Grimstad H, Moller A, Schei B, Janson PO (1999). Prevalence of physical and sexual abuse before and during pregnancy among Swedish couples. Acta Obstet Gynecol Scand.

[14] James L, Brody D, Hamilton Z (2013). Risk factors for domestic violence during pregnancy: a meta-analytic review. Violence Vict.

[15] Lukasse M, Schroll AM, Ryding EL, Campbell J, Karro H, Kristjansdottir H, Laanpere M, Steingrimsdottir T, Tabor A, Temmerman M, Van Parys AS, Wangel AM, Schei B (2014). Prevalence of emotional, physical and sexual abuse among pregnant women in six European countries. Acta Obstet Gynecol Scand.

[16] Taft A, O’Doherty L, Hegarty K, Ramsay J, Davidson L, Feder G (2013). Screening women for intimate partner violence in healthcare settings. Cochrane Database Syst Rev.

[17] Socialstyrelsen (2014), Att vilja se, vilja veta och att våga fråga. Vägledning för att öka förutsättningarna att upptäcka våldsutsatthet (In Swedish). http://www.socialstyrelsen.se/Lists/Artikelkatalog/Attachments/19568/2014-10-30.pdf. (ISBN:978-91-7555-224-8):3–42. In English: The National Board of Health and Welfare. 2014. Accessed 15 Nov 2014.

[18] Graviditetsregistret. Graviditetsregistret-mödrahälsovård-fosterdiagnostik- förlossning. (In Swedish). 2015:2–99. In English: Pregnancy Register. Pregnancy Register- Antenatal Care- Fetus diagnostic.delivery. Accessed 16 Sept 2015.

[19] Finnbogadottir H, Dykes AK (2012). Midwives’ awareness and experiences regarding domestic violence among pregnant women in southern Sweden. Midwifery.

[20] Campbell J, García-Moreno C, Sharps P (2004). Abuse During Pregnancy in Industrialized and Developing Countries. Violence Against Women.

[21] Antonovsky A (1993). The structure and properties of the sense of coherence scale. Soc Sci Med.

[22] Swahnberg IM, Wijma B (2003). The NorVold Abuse Questionnaire (NorAQ): validation of new measures of emotional, physical, and sexual abuse, and abuse in the health care system among women. Eur J Public Health.

[23] Cox JL, Holden JM, Sagovsky R (1987). Detection of Postnatal Depression. Development of the 10-item Edinburgh Postnatal depression Scale. Br J Psychiatry.

[24] Rubertsson C, Borjesson K, Berglund A, Josefsson A, Sydsjo G (2011). The Swedish validation of Edinburgh Postnatal Depression Scale (EPDS) during pregnancy. Nord J Psychiatry.

[25] Saunders JB, Aasland OG, Babor TF, de la Fuente JR, Grant M (1993). Development of the Alcohol Use Disorders Identification Test (AUDIT): WHO Collaborative Project on Early Detection of Persons with Harmful Alcohol Consumption-II. Addiction.

[26] Krug EG, Dahlberg LL, Mercy J, Zwi AB, Lozano R (2002). World report on violence and Health.

[27] WHO; Global Database on Body Mass Index. BMI classificatons. [http://apps.who.int/bmi/index.jsp?introPage=intro_3.html]. Accessed 22 Oct 2013.

[28] Ekelin M, Crang Svalenius E, Larsson AK, Nyberg P, Marsal K, Dykes AK (2009). Parental expectations, experiences and reactions, sense of coherence and grade of anxiety related to routine ultrasound examination with normal findings during pregnancy. Prenat Diagn.

[29] Lau Y (2005). Does pregnancy provide immunity from intimate partner abuse among Hong Kong Chinese women?. Soc Sci Med.

[30] Lundgren E (2004). The process of normalising violence.

[31] Finnbogadottir H, Dykes AK, Wann-Hansson C (2014). Struggling to survive for the sake of the unborn baby: a grounded theory model of exposure to intimate partner violence during pregnancy. BMC Pregnancy Childbirth.

[32] Yost NP, Bloom SL, McIntire DD, Leveno KJ (2005). A prospective observational study of domestic violence during pregnancy. Obstet Gynecol.

[33] Edin KE, Dahlgren L, Lalos A, Hogberg U (2010). “Keeping up a front”: narratives about intimate partner violence, pregnancy, and antenatal care. Violence Against Women.

[34] Engnes K, Liden E, Lundgren I: Experiences of being exposed to intimate partner violence during pregnancy. Int J Qual Stud Health Well-being 2012, 7. doi:10.3402/qhw.v7i0.11199. Epub 2012 Mar 15. 10.3402/qhw.v7i0.11199 PMC3313778 22468147

[35] Lutz KF (2005). Abused pregnant women’s interactions with health care providers during the childbearing year. J Obstet Gynecol Neonatal Nurs.

[36] Sorbo MF, Grimstad H, Bjorngaard JH, Schei B, Lukasse M (2013). Prevalence of sexual, physical and emotional abuse in the Norwegian mother and child cohort study. BMC Public Health.

[37] Devries KM, Kishor S, Johnson H, Stockl H, Bacchus LJ, Garcia-Moreno C, Watts C (2010). Intimate partner violence during pregnancy: analysis of prevalence data from 19 countries. Reprod Health Matters.

[38] Garcia-Moreno C, Jansen H, Ellsberg M, Heise L, Watts C (2005). WHO Multi-Country study on women’s health and domestic violence against women.

[39] Finnbogadottir H, Dejin-Karlsson E, Dykes AK (2011). A multi-centre cohort study shows no association between experienced violence and labour dystocia in nulliparous women at term. BMC Pregnancy Childbirth.

[40] Gazmararian JA, Lazorick S, Spitz AM, Ballard TJ, Saltzman LE, Marks JS (1996). Prevalence of violence against pregnant women. JAMA.

[41] Campbell JC, Campbell DW (1996). Cultural competence in the care of abused women. J Nurse Midwifery.

[42] Regeringskansliets rättsdatabaser, SFS (2001:453). 5 kap 11 §. Lag (2012:776) (in Swedish). http://www.notisum.se/rnp/sls/LAG/20010453.htm. In English: Government Office: Law for Social services (2001:453). 5 Chapter 11 §.Law (2012:776). Accessed 25 March 2014.

[43] Regeringskansliets rättsdatabaser, Patientsäkerhetslag (2010:659).6 kap. 5 § (in Swedish). http://www.notisum.se/rnp/sls/lag/20100659.htm. In English: Government Office: Law for patient safety (2010:659).6 Chapter. 5 §. Accessed 25 March 2014.

[44] Regeringskansliets rättsdatabaser, Tystnadsplikt & Sekretess (2010:659). 6 kap.12–16 § (in Swedish). http://www.notisum.se/rnp/sls/lag/20100659.htm. In English: Government Office: Professional secrecy & confidentiality (2010:659).6 Chapter. 12–16 §. Accessed 25 March 2014.

[45] American Psychiatric Association (2013). Diagnostic and Statistical Manual of Mental Disorders.

[46] Wickberg B, Hwang CP (1996). The Edinburgh Postnatal Depression Scale: validation on a Swedish community sample. Acta Psychiatr Scand.

[47] Antonovsky A (1991). Unraveling the mystery of health: how people manage stress and stay well.

[48] Wijma B, Schei B, Swahnberg K, Hilden M, Offerdal K, Pikarinen U, Sidenius K, Steingrimsdottir T, Stoum H, Halmesmaki E, Nordic cross-sectional study (2003). Emotional, physical, and sexual abuse in patients visiting gynaecology clinics: a Nordic cross-sectional study. Lancet.

[49] World Health Organization (2001). Putting women first: Ethical and safety recommendations for research on domestic violence against women.

[50] World Medical Association Declaration of Helsinki. Ethical Principles for Medical Research Involving Human Subjects. [http://www.wma.net/en/30publications/10policies/b3/]. Accessed 30 Oct 2013. 10.1001/jama.2013.281053 24141714

